# Parameter Identifiability and Redundancy: Theoretical Considerations

**DOI:** 10.1371/journal.pone.0008915

**Published:** 2010-01-27

**Authors:** Mark P. Little, Wolfgang F. Heidenreich, Guangquan Li

**Affiliations:** 1 Department of Epidemiology and Public Health, Imperial College Faculty of Medicine, London, United Kingdom; 2 Institut für Strahlenschutz, Helmholtz Zentrum München, German Research Center for Environmental Health, Ingolstädter Landstrasse, Neuherberg, Germany; University of East Piedmont, Italy

## Abstract

**Background:**

Models for complex biological systems may involve a large number of parameters. It may well be that some of these parameters cannot be derived from observed data via regression techniques. Such parameters are said to be unidentifiable, the remaining parameters being identifiable. Closely related to this idea is that of redundancy, that a set of parameters can be expressed in terms of some smaller set. Before data is analysed it is critical to determine which model parameters are identifiable or redundant to avoid ill-defined and poorly convergent regression.

**Methodology/Principal Findings:**

In this paper we outline general considerations on parameter identifiability, and introduce the notion of weak local identifiability and gradient weak local identifiability. These are based on local properties of the likelihood, in particular the rank of the Hessian matrix. We relate these to the notions of parameter identifiability and redundancy previously introduced by Rothenberg (*Econometrica* **39** (1971) 577–591) and Catchpole and Morgan (*Biometrika* **84** (1997) 187–196). Within the widely used exponential family, parameter irredundancy, local identifiability, gradient weak local identifiability and weak local identifiability are shown to be largely equivalent. We consider applications to a recently developed class of cancer models of Little and Wright (*Math Biosciences* **183** (2003) 111–134) and Little *et al.* (*J Theoret Biol* **254** (2008) 229–238) that generalize a large number of other recently used quasi-biological cancer models.

**Conclusions/Significance:**

We have shown that the previously developed concepts of parameter local identifiability and redundancy are closely related to the apparently weaker properties of weak local identifiability and gradient weak local identifiability—within the widely used exponential family these concepts largely coincide.

## Introduction

Models for complex biological systems may involve a large number of parameters. It may well be that some of these parameters cannot be derived from observed data via regression techniques. Such parameters are said to be unidentifiable or non-identifiable, the remaining parameters being identifiable. Closely related to this idea is that of redundancy, that a set of parameters can be expressed in terms of some smaller set. Before data is analysed it is critical to determine which model parameters are identifiable or redundant to avoid ill-defined and poorly convergent regression.

Identifiability in stochastic models has been considered previously in various contexts. Rothenberg [1] and Silvey [2] (pp. 50, 81) defined a set of parameters for a model to be identifiable if no two sets of parameter values yield the same distribution of the data. Catchpole and Morgan [3] considered identifiability and parameter redundancy and the relations between them in a general class of (exponential family) models. Rothenberg [1], Jacquez and Perry [4] and Catchpole and Morgan [3] also defined a notion of local identifiability, which we shall extend in the Analysis Section. [There is also a large literature on identifability in deterministic (rather than stochastic) models, for example the papers of Audoly *et al.* [5], and Bellu [6], which we shall not consider further.] Catchpole *et al.* [7] and Gimenez *et al.* [8] outlined use of computer algebra techniques to determine numbers of identifiable parameters in the exponential family. Viallefont *et al.* [9] considered parameter identifiability issues in a general setting, and outlined a method based on considering the rank of the Hessian for determining identifiable parameters; however, some of their claimed results are incorrect (as we outline briefly later). Gimenez *et al.* [8] used Hessian-based techniques, as well as a number of purely numerical techniques, for determining the number of identifiable parameters. Further general observations on parameter identifiability and its relation to properties of sufficient statistics are given by Picci [10], and a more recent review of the literature is given by Paulino and de Bragança Pereira [11].

In this paper we outline some general considerations on parameter identifiability. We shall demonstrate that the concepts of parameter local identifiability and redundancy are closely related to apparently weaker properties of weak local identifiability and gradient weak local identifiability that we introduce in the Analysis Section. These latter properties relate to the uniqueness of likelihood maxima and likelihood turning points within the vicinity of sets of parameter values, and are shown to be based on local properties of the likelihood, in particular the rank of the Hessian matrix. Within the widely-used exponential family we demonstrate that these concepts (local identifiability, redundancy, weak local identifiability, gradient weak local identifiability) largely coincide. We briefly consider applications of all these ideas to a recently developed general class of carcinogenesis models [12], [13], [14], presenting results that generalize those of Heidenreich [15] and Heidenreich *et al.* [16] in the context of the two-mutation cancer model [17]. These are outlined in the later parts of the Analysis and the Discussion, and in more detail in a companion paper [12].

## Analysis

### General Considerations on Parameter Identifiability

As outlined in the Introduction, a general criterion for parameter identifiability has been set out by Jacquez and Perry [4]. They proposed a simple linearization of the problem, in the context of models with normal error. They defined a notion of local identifiability, which is that in a local region of the parameter space, there is a unique 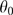 that fits some specified body of data, 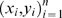, i.e. for which the model predicted mean 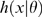 is such that the residual sum of squares:
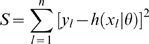
(1)has a unique minimum. We present here a straightforward generalization of this to other error structures. If the model prediction 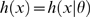 for the observed data 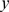 is a function of some vector parameters 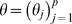 then in general it can be assumed, under the general equivalence of likelihood maximization and iteratively reweighted least squares for generalized linear models [18](chapter 2) that one is trying to minimize:
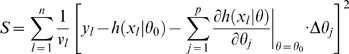
(2)where 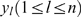 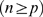 is the observed measurement (e.g., the numbers of observed cases in the case of binomial or Poisson models) at point 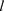 and the 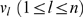 are the current estimates of variance at each point. This has a unique minimum in the perturbing 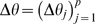 (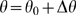) given by 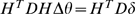, where 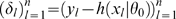, 
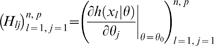
, 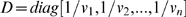, whenever 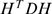 has full rank ( = 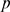).

More generally, suppose that the likelihood associated with observation 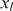 is 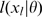 and let 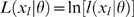. Then generalizing the least squares criterion (1) we now extend the definition of local identifiability to mean that there is at most one maximum of:
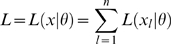
(3)in the neighborhood of any given 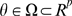. More formally:

#### Definitions 1

A set of parameters 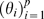 is identifiable if for any 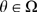 there are no 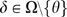 for which 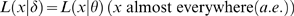. A set of parameters 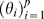 is locally identifiable if there exists a neighborhood 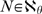 such that for no 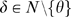 is 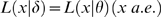. A set of parameters 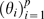 is weakly locally identifiable if there exists a neighborhood 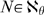 and data 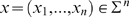 such that the log-likelihood 
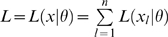
 is maximized by at most one set of 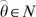. If 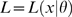 is 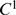 as a function of 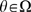 a set of parameters 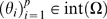 is gradient weakly locally identifiable if there exists a neighborhood 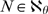 and data 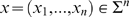 such that 
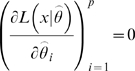
 (i.e., 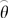 is a turning point of 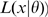) for at most one set of 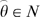.

Our definitions of identifiability and local identifiability coincide with those of Rothenberg [1], Silvey [2](pp. 50, 81) and Catchpole and Morgan [3]. Rothenberg [1] proved that if the Fisher information matrix, 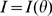, in a neighborhood of 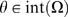 is of constant rank and satisfies various other more minor regularity conditions, then 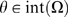 is locally identifiable if and only if 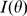 is non-singular. Clearly identifiability implies local identifiability, which in turn implies weak local identifiability. By the Mean Value Theorem [19](p. 107) gradient weak local identifiability implies weak local identifiability. Heuristically, (gradient) weak local identifiability happens when:
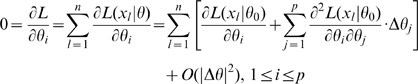
(4)and in general this system of 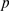 equations has a unique solution in 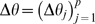 in the neighborhood of 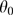 (assumed 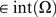) whenever 
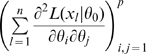
 has full rank ( = 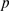). This turns out to be (nearly) the case, and will be proved later (Corollary 2). More rigorously, we have the following result.

#### Theorem 1

Suppose that the log-likelihood 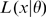 is 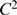 as a function of the parameter vector 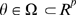, for all 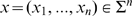.

- Suppose that for some 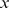 and 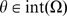 it is the case that 
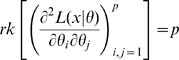
. Then turning points of the likelihood in the neighborhood of 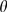 are isolated, i.e., there is an open neighborhood 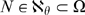 for which there is at most one 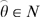 that satisfies 
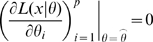
.
- Suppose that for some 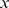 and 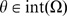 it is the case that 
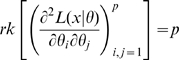
 then local maxima of the likelihood in the neighborhood of 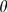 are isolated, i.e., there is an open neighborhood 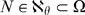 for which there is at most one 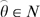 that is a local maximum of 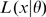.
- Suppose that for some 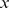 and all 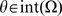 it is the case that 
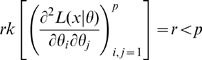
 then all local maxima of the likelihood in 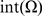 are not isolated, as indeed are all 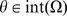 for which 
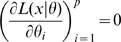
.

We prove this result in Text S1 Section A. As an immediate consequence we have the following result.

#### Corollary 1

For a given 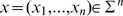, a sufficient condition for the likelihood (3) to have at most one maximum and one turning point in the neighborhood of a given 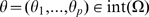 is that 
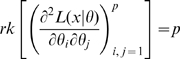
. In particular, if this condition is satisfied 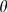 is gradient weakly locally identifiable (and therefore weakly locally identifiable). (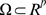 is the parameter space.)

That this condition is not necessary is seen by consideration of the likelihood 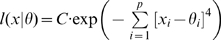, where 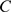 is chosen so that this has unit mass. Then 
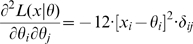
 which has rank 0 at 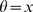 and a unique maximum there. In particular, this shows that the result claimed by Viallefont *et al.* [9](proposition 2, p. 322) is incorrect.

#### Definitions 2

A subset of parameters 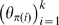 (for some permutation 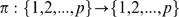) is weakly maximal (respectively weakly gradient maximal) if for any permissible fixed 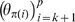 (such that 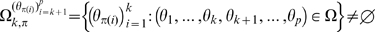) 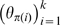 is weakly locally identifiable (respectively gradient weakly locally identifiable) at that point, but that this is not the case for any larger number of parameters. A subset of parameters 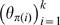 is strongly maximal (respectively strongly gradient maximal) if for any permissible fixed 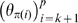 and any open 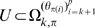, 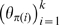 restricted to the set 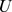 is weakly maximal (respectively weakly gradient maximal), i.e., all 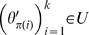 are weakly maximal (respectively weakly gradient maximal).

From this it easily follows that a strongly (gradient) maximal set of parameters 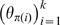 is *a fortiori* weakly (gradient) maximal at all points 
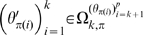
 for any permissible 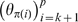. Assume now that 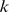 of the 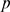 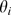 are a weakly maximal set of parameters. So for some permutation 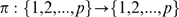 and for any permissible fixed 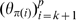 and any 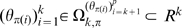 there is an open neighborhood 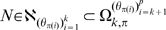 and some data 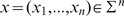 for which 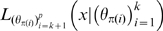 is maximized by at most one set of 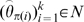, but that this is not the case for any larger number of parameters. Assume that 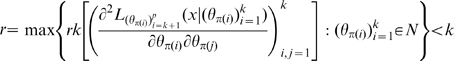. If 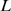 is 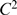 as a function of 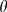 then it follows easily that 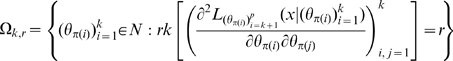 must be an open non-empty subset of 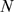. By Theorem 1 (iii) any 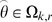 which maximizes 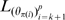 in 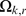 cannot be isolated, a contradiction (unless there are no maximizing 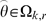). Therefore, either there are no maximizing 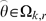 or for at least one 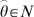 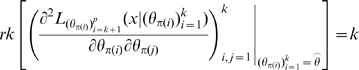. This implies that 
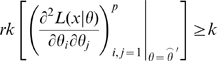
, where 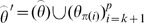 in the obvious sense.

Assume now that the 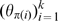 are strongly maximal. Suppose that for some 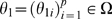 and some 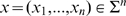 it is the case that 
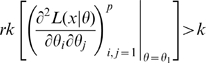
. Because 
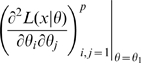
 is symmetric, there is a permutation 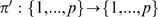 for which 
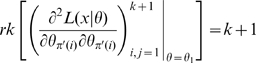
 [20](p. 79). If 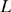 is 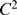 as a function of 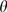 this will be the case in some open neighborhood 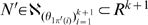. By Theorem 1 (ii) this implies that the parameters 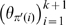 have at most one maximum in 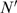, so that 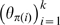 is not a strongly maximal set of parameters in 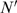. With small changes everything above also goes through with “weakly gradient maximal” substituted for “weakly maximal” and “strongly gradient maximal” substituted for “strongly maximal”. Therefore we have proved the following result.

#### Theorem 2

Let 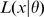 be 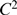 as a function of 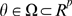 for all 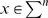.

- If there is a weakly maximal (respectively weakly gradient maximal) subset of 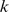 parameters, 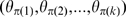 (for some permutation 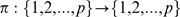), and for fixed 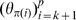 and some 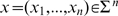 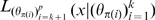 has a maximum (respectively turning point) on the set of 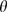 where 
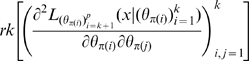
 is maximal then 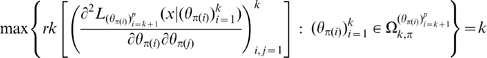 and 
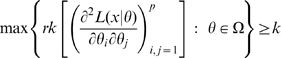
.
- If there is a strongly maximal (respectively strongly gradient maximal) subset of 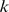 parameters, 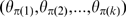 (for some permutation 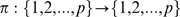) then 
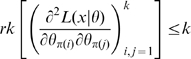
 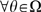.

All further results in this Section assume that the model is a member of the exponential family, so that if the observed data 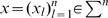 then the log-likelihood is given by 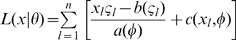 for some functions 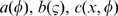. We assume that the natural parameters 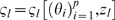 are functions of the model parameters 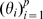 and some auxiliary data 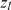, but that the scaling parameter 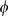 is not. Let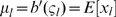, so that 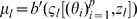. In all that follows we shall assume that the function 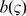 is 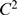. The following definition was introduced by Catchpole and Morgan [3].

#### Definition 3

With the above notation, a set of parameters 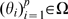 is parameter redundant for an exponential family model if 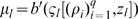 can be expressed in terms of some strictly smaller parameter vector 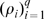 (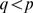). Otherwise, the set of parameters 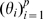 is parameter irredundant or full rank.

Catchpole and Morgan [3] proved (their Theorem 1) that a set of parameters is parameter redundant if and only if 
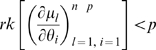
. They defined full rank models to be essentially full rank if 
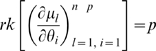
 for every 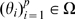; if 
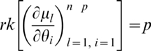
 only for some 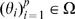 then the parameter set is conditionally full rank. They also showed (their Theorem 3) that if 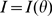 is the Fisher information matrix then 
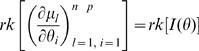
, and that parameter redundancy implies lack of local identifiability; indeed their proof of Theorems 2 and 4 showed that there is also lack of weak local identifiability (respectively gradient weak local identifiability) for all 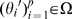 which for some 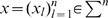 are local maxima (respectively turning points) of the likelihood.

Assume that 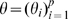 are an essentially full rank set of parameters for the model. From the above result for every 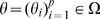 
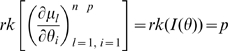
. Therefore, since 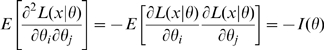 is of full rank and so negative definite, so by the strong law of large numbers we can choose 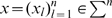 so that the same is true of 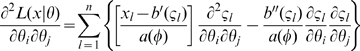. This implies that on some 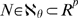 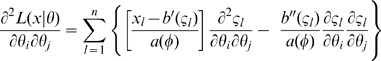 is of full rank, and therefore by Corollary 1 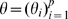 is (gradient) weakly locally identifiable. Furthermore, the above argument shows that if 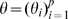 are a conditionally full rank set of parameters then on the (open) set 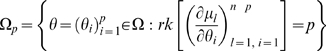 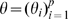 is gradient weakly locally identifiable. We have therefore proved:

#### Theorem 3

Let 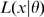 belong to the exponential family and be 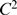 as a function of 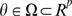 for all 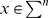.

- If the parameter set 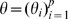 is parameter redundant then it is not locally identifiable, and is not weakly locally identifiable (respectively gradient weakly locally identifiable) for all 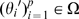 which for some 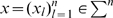 are local maxima (respectively turning points) of the likelihood.
- If the parameter set 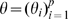 is of essentially full rank then for some 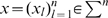 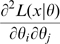 is of full rank and therefore 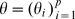 is gradient weakly locally identifiable (and so weakly locally identifiable) for all 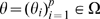.
- If the parameter set 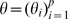 is of conditionally full rank then it is gradient weakly locally identifiable on the open set 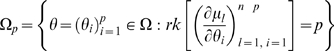.

Remarks: It should be noted that part (i) of this generalizes part (i) of Theorem 4 of Catchpole and Morgan [3], who proved that if a model is parameter redundant then it is not locally identifiable. However, some components of part (ii) (that being essentially full rank implies gradient weak local identifiability) is weaker than the other result, proved in part (ii) of Theorem 4 of Catchpole and Morgan [3], namely that if a model is of essentially full rank it is locally identifiable. As noted by Catchpole and Morgan [3] (pp. 193–4), there are exponential-family models that are conditionally full rank, but not locally identifiable, so part (iii) is about as strong a result as can be hoped for.

From Theorem 3 we deduce the following.

#### Corollary 2

Let 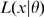 belong to the exponential family and be 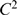 as a function of 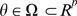 for all 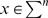. Then

- If for some subset of parameters 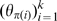 and some 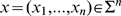 it is the case that 
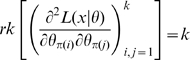
 then this subset is gradient weakly locally identifiable at this point.
- If a subset of parameters 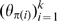 is weakly locally identifiable and for some 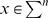 this point is a local maximum of the likelihood then it is parameter irredundant, i.e., of full rank, so 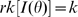, so that for some 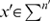 
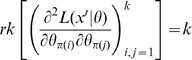
. In particular, if this holds for all 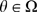 then parameter irredundancy, local identifiability, gradient weak local identifiability and weak local identifiability are all equivalent.

#### Proof

This is an immediate consequence of the remarks after Definition 1, Corollary 1, Theorem 3 (i) and Theorems 1 and 3 of Catchpole and Morgan [3]. **QED**.

Remarks: (i) By the remarks preceding Theorem 3 the conditions of part (i) (that for some 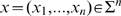 it is the case that 
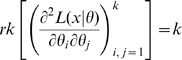
) are automatically satisfied if 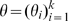 are an essentially full rank set of parameters for the model.

(ii) Assume the model is constructed from a stochastic cancer model embedded in the exponential family, in the sense outlined in Text S1 Section B, so that the natural parameters 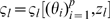 are functions of the model parameters 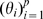 and some auxiliary data 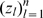, and the means are given by 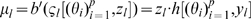, where 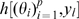 is the cancer hazard function. In this case, as shown in Text S1 Section B, 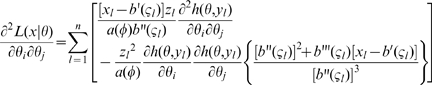. The second term inside the summation 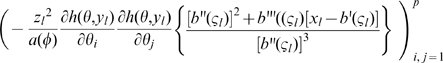 is a rank 1 matrix and can be made small in relation to the first term, e.g., by making 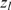 small. Therefore finding data 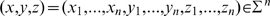 for which 
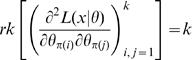
 is equivalent to finding data for which 
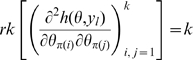
, or by the result of Dickson [20](p. 79) for which 
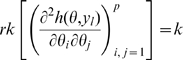
.

### Hessian vs Fisher Information Matrix as a Method of Determining Redundancy and Identifiability in Generalised Linear Models

We, as with Catchpole and Morgan [3], emphasise use of the Hessian of the likelihood rather than the Fisher information matrix considered by Rothenberg [1]. In the context of GLMs, we have 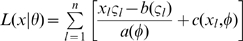 and 
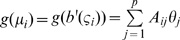
 for some link function 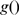 and fixed matrix 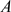. We define 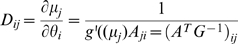 where 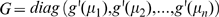. Theorem 1 of Catchpole and Morgan [3] states that a model is parameter irredundant if and only if 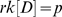. The score vector is given by 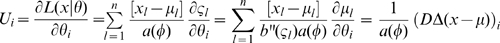 where 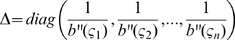. The Fisher information is therefore given by 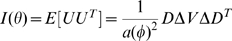 where 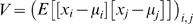 is the data variance. Theorem 1 of Rothenberg [1] states that a model is locally identifiable if and only if 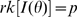. As above (Corollary 2 (ii)), heuristically parameter irredundancy, local identifiability, gradient weak local identifiability and weak local identifiability are all equivalent and occur whenever 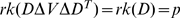. Clearly evaluating the rank of 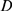 is generally much easier than that of 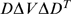. Catchpole and Morgan [3] demonstrate use of Hessian-based methods to estimate parameter redundancy in a class of capture-recapture models.

However, for certain applications, both the Fisher information and the Hessian must be employed, as we now outline. Assume that the model is constructed from a stochastic cancer model embedded in an exponential family model in the sense outlined in Text S1 Section B. The key to showing that such an embedded model has no more than 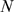 irredundant parameters is to construct (as is done in Little *et al.* [12]) some scalar functions 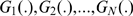 such that the cancer hazard function 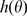 can be written as 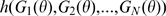. Since the cancer model is embedded in a member of the exponential family (in the sense outlined in Text S1 Section B) the same will be true of the total log-likelihood 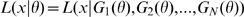. By means of the Chain Rule we obtain 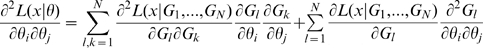, so that the Fisher information matrix is given by:
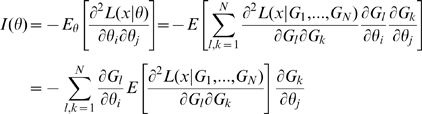
(5)which therefore has rank at most 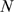. Therefore by Corollary 2 there can be at most 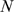 irredundant parameters, or indeed (gradient) weak locally identifiable parameters. [A similar argument shows that if one were to reparameterise (via some invertible 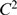 mapping 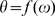) then the embedded log-likelihood 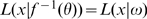 associated with 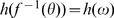 must also have Fisher information matrix of rank at most 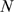.] By remark (ii) after Corollary 2, to show that a subset of cardinality 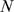 of the parameters 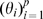 is (gradient) weak locally identifiable parameters, requires that one show that 
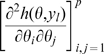
 has rank at least 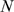 for some 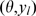. This is the approach adopted in the paper of Little *et al.* [12].

## Discussion

In this paper we have introduced the notions of weak local identifiability and gradient weak local identifiability, which we have related to the notions of parameter identifiability and redundancy previously introduced by Rothenberg [1] and Catchpole and Morgan [3]. In particular we have shown that within the exponential family models parameter irredundancy, local identifiability, gradient weak local identifiability and weak local identifiability are largely equivalent.

The slight novelty of our approach is that the notions of weak local identifiability and gradient weak local identifiability that we introduce are related much more to the Hessian of the likelihood rather than the Fisher information matrix that was considered by Rothenberg [1]. However, in practice, the two approaches are very similar; Catchpole and Morgan [3] used the Hessian of the likelihood, as do we, because of its greater analytic tractability. The use of this approach is motivated by the application, namely to determine identifiable parameter combinations in a large class of stochastic cancer models, as we outline at the end of the Analysis Section. In certain applications the Fisher information may be best for estimating the upper bound to the number of irredundant parameters, but the Hessian may be best for estimating the lower bound of this quantity.

In the companion paper of Little *et al.* [12] we consider the problem of parameter identifiability in a particular class of stochastic cancer models, those of Little and Wright [13] and Little *et al.* [14]. These models generalize a large number of other quasi-biological cancer models, in particular those of Armitage and Doll [21], the two-mutation model [17], the generalized multistage model of Little [22], and a recently developed cancer model of Nowak *et al.* [23] that incorporates genomic instability. These and other cancer models are generally embedded in an exponential family model in the sense outlined in Text S1 Section B, in particular when cohort data are analysed using Poisson regression models, e.g., as in Little *et al.* [13], [14], [24]. As we show at the end of the Analysis Section, proving (gradient) weak local identifiability of a subset of cardinality 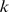 of the parameters 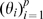 can be done by showing that for this subset of parameters 
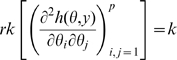
 where 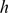 is the cancer hazard function. Little *et al.* [12] demonstrate (by exhibiting a particular parameterization) that there is redundancy in the parameterization for this model: the number of theoretically estimable parameters in the models of Little and Wright [13] and Little *et al.* [14] is at most two less than the number that are theoretically available, demonstrating (by Corollary 2) that there can be no more than this number of irredundant parameters. Two numerical examples suggest that this bound is sharp – we show that the rank of the Hessian, 
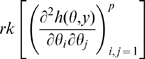
, is two less than the row dimension of this matrix. This result generalizes previously derived results of Heidenreich and others [15], [16] relating to the two-mutation model.

## Supporting Information

Text S1 (0.33 MB DOC)
