# Supplementary material for: Parameter Identifiability and Redundancy: Theoretical Considerations
Source: PLoS One. 2010 Jan 27;5(1):e8915. doi: 10.1371/journal.pone.0008915 (PMC2811744; doi:10.1371/journal.pone.0008915)
Supplement: Text S1 — (0.33 MB DOC) [file pone.0008915.s001.doc]

**Supplementary material A.**

**Proof of Theorem 1**

In this Section we outline a proof of Theorem 1 in the main text. To prove this result we need the following lemma of Rudin [19](p.229).

**Lemma A1.** Suppose $m,n,r$ are non-negative integers s.t .$m\geq r$, $n\geq r$ and $F$ is a $C^{1}$ function $E\subset R^{n}\to R^{m}$ where $E$ is an open set. Suppose that $rk(F'(x))=r$ $\forall x\in E$. Fix $a\in E$ and put $A=F'(a)$, and let $Y_{1}=A(R^{n})$ and let $P:R^{m}\to R^{m}$ be a linear projection operator ($P^{2}=P$) s.t. $Y_{1}=P(R^{m})$ and $Y_{2}=null(P)$. Then $\exists U,V\subset R^{n}$, open sets and a bijective $C^{1}$ function $H:V\to U$ whose inverse is also $C^{1}$ and s.t. $F(H(x))=Ax+\phi(Ax),$ $\forall x\in V$ where $\phi:AV\subset Y_{1}\to Y_{2}$ is a $C^{1}$ function.

We now restate Theorem 1 here.

**Theorem A2.** Suppose that the log-likelihood $L(x|\theta)$ is $C^{2}$ as a function of the parameter vector $\theta\in\Omega\subset R^{p}$, and for all$x=(x_{1},...,x_{n})\in\Sigma^{n}$.

1. Suppose that for some $x$ and $\theta\in\mathrm{int}(\Omega)$ it is the case that $rk\left[\left(\frac{\partial^{2}L(x|\theta)}{\partial\theta_{i}\partial\theta_{j}}\right)_{i,j=1}^{p}\right]=p$. Then turning points of the likelihood in the neighborhood of $\theta$ are isolated, i.e., there is an open neighborhood $N\in\aleph_{\theta}\subset\Omega$ for which there is at most one that satisfies .
2. Suppose that for some $x$ and $\theta\in\mathrm{int}(\Omega)$ it is the case that $rk\left[\left(\frac{\partial^{2}L(x|\theta)}{\partial\theta_{i}\partial\theta_{j}}\right)_{i,j=1}^{p}\right]=p$ then local maxima of the likelihood in the neighborhood of $\theta$ are isolated, i.e., there is an open neighborhood $N\in\aleph_{\theta}\subset\Omega$ for which there is at most one that is a local maximum of $L(x|\theta)$.
3. Suppose that for some $x$ and all $\theta\in\mathrm{int}(\Omega)$ it is the case that $rk\left[\left(\frac{\partial^{2}L(x|\theta)}{\partial\theta_{i}\partial\theta_{j}}\right)_{i,j=1}^{p}\right]=r<p$ then all local maxima of the likelihood in $\mathrm{int}(\Omega)$ are not isolated, as indeed are all $\theta\in\mathrm{int}(\Omega)$ for which $\left(\frac{\partial L(x|\theta)}{\partial\theta_{i}}\right)_{i=1}^{p}=0$.

**Proof:**

(i) Let $F:\Omega\subset R^{p}\to R^{p}$ be defined by $F(\theta_{1},\theta_{2},...,\theta_{p})=\left(\frac{\partial L(x|\theta)}{\partial\theta_{1}},\frac{\partial L(x|\theta)}{\partial\theta_{2}},...,\frac{\partial L(x|\theta)}{\partial\theta_{p}}\right)$. Since $L$ is $C^{2}$, $F$ is $C^{1}$ on $\mathrm{int}(\Omega)\subset R^{p}$. By assumption $\frac{\partial F(\theta)_{i}}{\partial\theta_{j}}=\left(\frac{\partial^{2}L(x|\theta)}{\partial\theta_{j}\partial\theta_{i}}\right)_{i,j=1}^{p}$ is of full rank at $\theta$. By the inverse function theorem [19](pp.221-223) there are open $N,M\subset R^{p}$ such that $\theta\in N$ and a $C^{1}$ bijective function $G:M\to N$ such that for all . In particular there can be at most a single for which . **QED.**

(ii) By (i) there is an open neighborhood $N\in\aleph_{\theta}\subset\Omega$ for which if is such that then for $\left.\left(\frac{\partial L(x|\theta)}{\partial\theta_{i}}\right)_{i=1}^{p}\right|_{\theta=\theta'}\neq0$. Suppose now that is a local maximum of $L(x|\theta)$. Any member of this neighborhood other than cannot be a turning point, and so by the Mean Value Theorem (Rudin 1976, p.107) cannot be a local maximum. **QED.**

(iii) Let $F:\Omega\subset R^{p}\to R^{p}$ be defined by $F(\theta_{1},\theta_{2},...,\theta_{p})=\left(\frac{\partial L(x|\theta)}{\partial\theta_{1}},\frac{\partial L(x|\theta)}{\partial\theta_{2}},...,\frac{\partial L(x|\theta)}{\partial\theta_{p}}\right)$. Since $L$ is

$C^{2}$, $F$ is $C^{1}$ on $\mathrm{int}(\Omega)\subset R^{p}$. By assumption $rk(F)=rk\left[\left(\frac{\partial^{2}L(x|\theta)}{\partial\theta_{i}\partial\theta_{j}}\right)_{i,j=1}^{p}\right]=r$ for all $\theta\in\mathrm{int}(\Omega)\subset R^{p}$. Suppose that $\theta_{0}\in\mathrm{int}(\Omega)$ is a local maximum of $L$. Let $A=\left.\frac{\partial F}{\partial\theta}\right|_{\theta=\theta_{0}}:R^{p}\to R^{p}$ ($A\in L(R^{p},R^{p})$), and choose some arbitrary projection $P\in L(R^{p},R^{p})$s.t. $P(R^{p})=Y_{1}=A(R^{p})$, and let $Y_{2}=null(P)$. By Lemma A1 there are open sets $U,V\subset R^{p}$ with $\theta_{0}\in U\subset\mathrm{int}(\Omega)$ and a bijective $C^{1}$ mapping with $C^{1}$ inverse $H:V\to U$ s.t. $F(y)=AH^{-1}y+\phi(AH^{-1}y),$ $\forall y\in V$ where $\phi:AV\subset Y_{1}\to Y_{2}$ is a $C^{1}$ function.

Since $\theta_{0}\in\mathrm{int}(\Omega)$ is a local maximum of $L(x;\theta)$, by the Mean Value Theorem [19](p.107) . Now choose some non-trivial vector and define a function, as we can, on some interval $\delta:(-\varepsilon,\varepsilon)\to R^{p}$ by . Because $H:V\to U$ is bijective and is non-trivial $\delta(t)=\delta(t')\Leftrightarrow t=t'$. Also, it is the case that:

(A1)

Define $G:(-\varepsilon,\varepsilon)\to R$ by $G(t)=L(\delta(t))=L((\delta_{1}(t),\delta_{2}(t),...,\delta_{n}(t)))$. By the chain rule [19](p.215) . Finally, by the Mean Value Theorem [19](p.107) must be constant; in particular $L(x|\delta(t))=L(x|\delta(0))=L(x|\theta_{0})$ $\forall t\in(-\varepsilon,\varepsilon)$ and so all points $\delta(t)$ must also be local maxima of $L(x|\theta)$. Therefore $\theta_{0}$ is not an isolated local maximum. Since all we used about $\theta_{0}\in\mathrm{int}(\Omega)$ was that $F(\theta_{0})=0$ $F((\theta_{0i})_{i=1}^{p})=\left(\frac{\partial L(x|\theta_{0})}{\partial\theta_{01}},\frac{\partial L(x|\theta_{0})}{\partial\theta_{02}},...,\frac{\partial L(x|\theta_{0})}{\partial\theta_{0p}}\right)=0$, the above argument also shows that turning points cannot be isolated: $F(\delta(t))=0$. **QED.**

**Supplementary material B.**

**Specification of embedded exponential family model**

In this Section we outline the specification of an embedding of a stochastic cancer model in a general class of statistical models, the so-called exponential family [18]. This is often done in fitting cancer models to epidemiological and biological data (e.g., see references [12, 13, 14, 24]). Recall that a model is a member of the exponential family if the observed data $x=(x_{l})_{l=1}^{n}\in\sum^{n}$ is such that the log-likelihood is given by $L(x|\theta)=\sum_{l=1}^{n} \left[\frac{x_{l}\varsigma_{l}-b(\varsigma_{l})}{a(\varphi)}+c(x_{l},\varphi)\right]$ for some functions $a(\varphi),b(\varsigma),c(x,\varphi)$. We assume that the natural parameters $\varsigma_{l}=\varsigma_{l}[(\theta_{i})_{i=1}^{p},z_{l}]$ are functions of the model parameters $(\theta_{i})_{i=1}^{p}$ and some auxiliary data $(z_{l})_{l=1}^{n}$, and that $\mu_{l}=b'(\varsigma_{l}[(\theta_{i})_{i=1}^{p},z_{l}])=z_{l}\cdot h[(\theta_{i})_{i=1}^{p},y_{l}]$. Here $h[(\theta_{i})_{i=1}^{p},y_{l}]$ is the cancer hazard function (for example, that of Little *et al.* [14], as also specified in the main text and in Text S1 Section B of Little *et al.* [12]), $(y_{l})_{l=1}^{n}$ are some further auxiliary data, and we assume that the $(z_{l})_{l=1}^{n}$ are all non-zero. [Note: this is not necessarily a generalized linear model (GLM).] In this case it is seen that

$\frac{\partial^{2}L(x|\theta)}{\partial\theta_{i}\partial\theta_{j}}=\sum_{l=1}^{n} \left[\begin{matrix} \frac{[x_{l}-b'(\varsigma_{l})]z_{l}}{a(\varphi)b''(\varsigma_{l})}\frac{\partial^{2}h(\theta,y_{l})}{\partial\theta_{i}\partial\theta_{j}} \\ -\frac{z_{l}^{2}}{a(\varphi)}\frac{\partial h(\theta,y_{l})}{\partial\theta_{i}}\frac{\partial h(\theta,y_{l})}{\partial\theta_{j}}\left\{\frac{[b''(\varsigma_{l})]^{2}+b'''(\varsigma_{l})[x_{l}-b'(\varsigma_{l})]}{[b''(\varsigma_{l})]^{3}}\right\} \end{matrix}\right]$ (B1)

so that the Fisher information matrix is given by

$I(\theta)_{ij}=-E_{\theta}\left[\frac{\partial^{2}L(x|\theta)}{\partial\theta_{i}\partial\theta_{j}}\right]=\frac{1}{a(\varphi)}\sum_{l=1}^{n} \frac{z_{l}^{2}}{b''(\varsigma_{l})}\frac{\partial h(\theta,y_{l})}{\partial\theta_{i}}\frac{\partial h(\theta,y_{l})}{\partial\theta_{j}}$ (B2)
